# Arachnoid Web With Atypical Imaging Findings and Clinical Manifestations: A Case Report

**DOI:** 10.7759/cureus.81308

**Published:** 2025-03-27

**Authors:** Eijiro Onishi, Yushi Sakamoto, Sadaki Mitsuzawa, Daiki Sako, Tadashi Yasuda

**Affiliations:** 1 Orthopedic Surgery, Kobe City Medical Center General Hospital, Kobe, JPN; 2 Orthopedic Surgery, Hyogo Prefectural Amagasaki General Medical Center, Amagasaki, JPN

**Keywords:** brown-sequard syndrome, magnetic resonance image, scalpel sign, spinal arachnoid web, ultrasonography

## Abstract

Spinal arachnoid web (SAW) is a rare intradural lesion that can result in spinal cord compression and myelopathy. This report describes a case of SAW with atypical imaging findings and clinical manifestations in a 63-year-old male patient who presented with progressive lower limb paresthesia and left-dominant muscle weakness. The patient also exhibited temperature and pain sensory disturbances in the right leg. Magnetic resonance imaging (MRI) revealed spinal cord atrophy, intramedullary hyperintensity, and a dorsal flow void without the scalpel sign at the T7 level. Laminectomy and surgical resection of the SAW were performed. Intraoperative ultrasound revealed a SAW obstructing the flow of cerebrospinal fluid (CSF). The left and right sides of the posterior space were divided by the septum posticum, which moved pulsatile from side to side. Postoperative improvements in muscle strength, temperature, and pain sensation were observed; however, numbness and bladder dysfunction persisted. Diagnosing SAW is challenging in the absence of the scalpel sign; however, in cases of spinal cord atrophy and a dorsal CSF flow void on MRI, the presence of SAW should be considered. Brown-Sequard syndrome may result from asymmetric CSF pressure caused by the septum posticum.

## Introduction

Spinal arachnoid webs (SAWs) are rare and challenging-to-diagnose intradural lesions of the spinal cord and are often observed in the thoracic spinal cord of men in their fifth and sixth decades of life [1]. Thickening and scarring of the arachnoid tissue lead to localized cerebrospinal flow changes that compress the spinal cord from the posterior, resulting in spinal cord symptoms. Spinal cord compression frequently leads to the formation of a syrinx [1,2]. Symptoms associated with SAW primarily manifest as myelopathy, including sensory impairment, pain, lower limb muscle weakness, and bowel and bladder dysfunction [2-6]. Owing to the potential for a delayed initial diagnosis, treatment for this condition is often initiated after it has already progressed.

Directly visualizing SAWs by magnetic resonance imaging (MRI) and computed tomography (CT) is challenging, and the diagnosis is generally difficult. The scalpel sign, a scalpel-like deformation of the dorsal portion of the spinal cord caused by dorsal compression of the spinal cord due to SAW, is well known in imaging diagnosis and has been identified in many cases [1-3,7]. However, it is important to distinguish SAW from spinal cord herniation, a condition that presents with similar imaging findings [8].

Intraoperative ultrasound has been a reliable method for the definitive diagnosis of SAW and evaluation of the extension of the lesion. Furthermore, ultrasound has been shown to be a valuable tool in excluding other diseases, such as spinal cord herniation, and it may also be effective for evaluating cerebrospinal fluid (CSF) flow following SAW resection [1,2,9].

Herein, we report a case in which SAW was confirmed using intraoperative ultrasound in the absence of a clear scalpel sign on preoperative imaging. This is a rare case in which the patient presented with Brown-Sequard syndrome with left-right differences in the lower extremity symptoms, which may have been caused by differences in cerebrospinal pressure.

## Case presentation

Patient presentation

**Figure 1:**
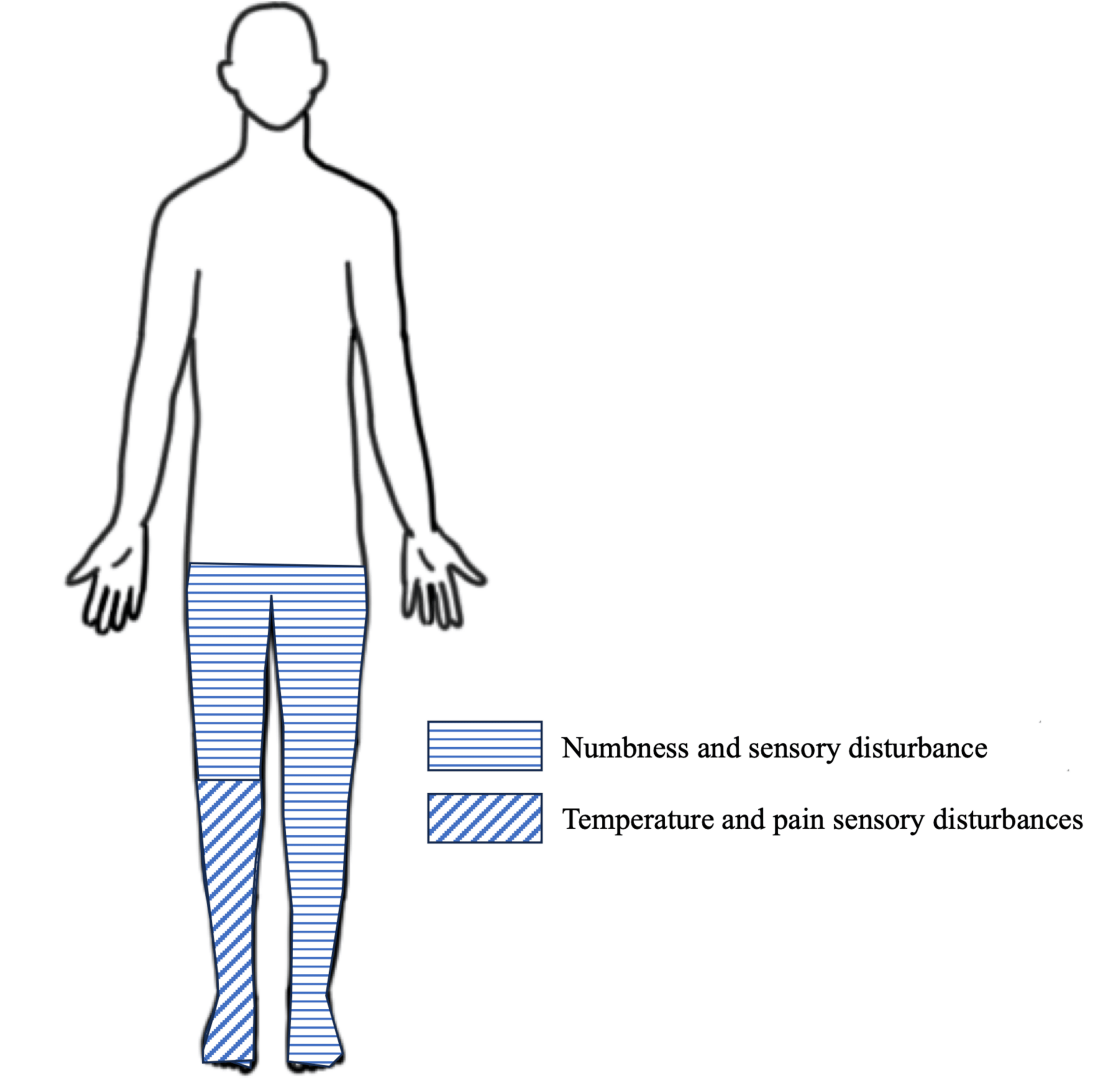
Distribution of sensory disturbance. The patient exhibited numbness and sensory disturbances in both lower limbs. Temperature and pain sensory disturbances were observed in the right lower leg but not in the left leg.

A 63-year-old male patient had previously undergone endoscopic decompression surgery for lumbar spinal canal stenosis at another medical institution approximately a decade ago. Five years ago, he began to develop numbness and sensory disturbances in both lower limbs. Two years later, the patient developed dysuria necessitating intermittent self-urination. One month prior, the patient presented to our hospital with walking difficulty due to muscle weakness in the lower extremities. The muscle strength grade of the lower extremities was 4 on the right and 2 on the left, with the left lower extremity exhibiting more impairment. He demonstrated a marked reduction in ambulation and bowel and bladder disturbances. Numbness and sensory disturbances were observed in both lower limbs, with more severe numbness in the right lower limb. He also exhibited temperature and pain sensory disturbances in the right lower leg but not in the left leg (Figure 1).

Symptoms progressed with muscle weakness, demonstrating gradual exacerbation. The patient had no history of spinal trauma.

Radiological findings

**Figure 2:**
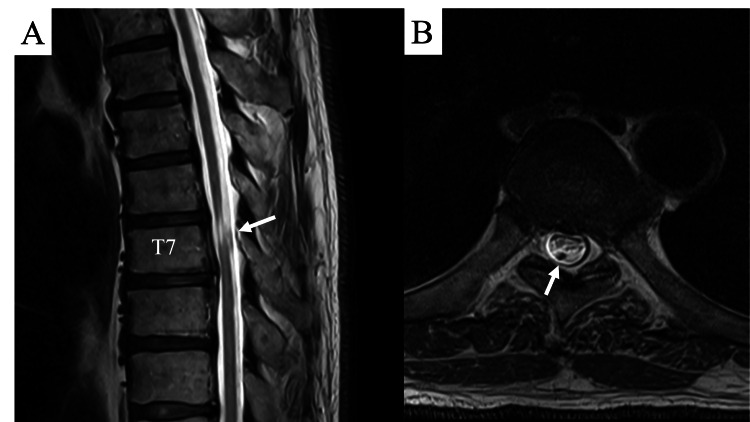
Magnetic resonance images. A: Magnetic resonance image showing atrophy of the spinal cord at the T7 level and a flow void (white arrow) dorsal to the atrophic spinal cord. B: Horizontal slice showed a flattened spinal cord and flow void (white arrow).

Magnetic resonance images revealed spinal cord atrophy and flattening at the T6/7 level and intramedullary hyperintensities on T2-weighted MRI, suggesting spinal cord compression from the dorsal side. However, no scalpel sign or syrinx was identified (Figure 2).

**Figure 3:**
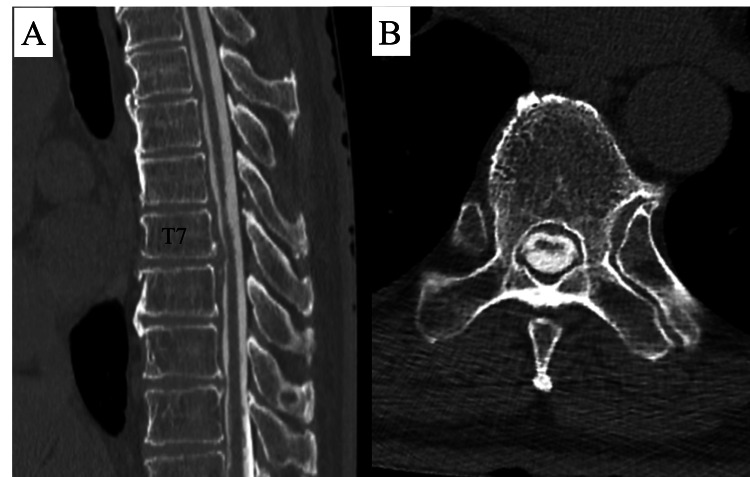
CT images of myelogram. A: Computed tomographic myelography demonstrates the atrophy and flattening of the spinal cord at T. B: A horizontal slice showed a hyperdense contrast accumulation posterior to the atrophied spinal cord.

An intradural flow void on MRI was also identified posterior to the spinal cord, where intramedullary hyperintensities were observed. Additionally, disc protrusions at the T6/7 and 7/8 levels were observed. Consequently, CT myelography results corroborated the MRI findings, indicating spinal cord atrophy, flattening, and mild anterior compression due to disc herniation. A horizontal slice of the CT scan at the T6/7 level revealed a slightly hyperdense contrast accumulation posterior to the atrophied spinal cord (Figure 3). Given the progressive nature of the symptoms, the surgical intervention was considered, with the differential diagnoses of spinal cord herniation, intervertebral disc herniation, intradural spinal cord tumor, and spinal arachnoid web.

Treatment and outcome

As a definitive diagnosis could not be established preoperatively, the surgical procedure was undertaken by considering the aforementioned differential diagnosis. The surgical procedure involved laminectomy of the sixth to eighth thoracic vertebrae and exposure of the dura mater. Posterior fusion was performed to circumvent the potential consequences of a herniated disc. Subsequent to laminectomy, the dura mater was examined under the microscope, but no evident abnormal findings were identified. Consequently, the decision was made to search for intradural lesions using ultrasound. Regarding the sagittal ultrasonographic findings, the arachnoid membrane was attached to the dorsal surface of the spinal cord at the T7 level, forming an arachnoid web, and CSF was obstructed at this site. The spinal cord was compressed anteriorly and thinned at the same site. A space anterior to the spinal cord for the CSF was observed, thereby ruling out the possibility of spinal cord herniation (Video 1) [8].

**Video 1:** Intraoperative ultrasonography image in the sagittal plane. Intraoperative ultrasonography image in the sagittal plane showing an arachnoid web. The spinal cord is compressed ventrally. The cerebrospinal fluid space is located anterior to the spinal cord.

Regarding the horizontal ultrasonographic findings (Video 2), horizontal sectioning at the T6 level, slightly more cranial than the arachnoid web, revealed increased CSF pressure in the dorsal spinal cord, indicating pulsatile anterior compression of the spinal cord. The left and right sides of the posterior space of the spinal cord were divided by the septum posticum, which moved in a pulsatile manner from side to side, suggesting a difference in cerebrospinal pressure between the left and right sides.

**Video 2:** Ultrasonography image in the horizontal plane. Intraoperative ultrasound in the horizontal plane (the right side of the screen is on the left side). The septum separates the posterior CSF flow space.

**Figure 4:**
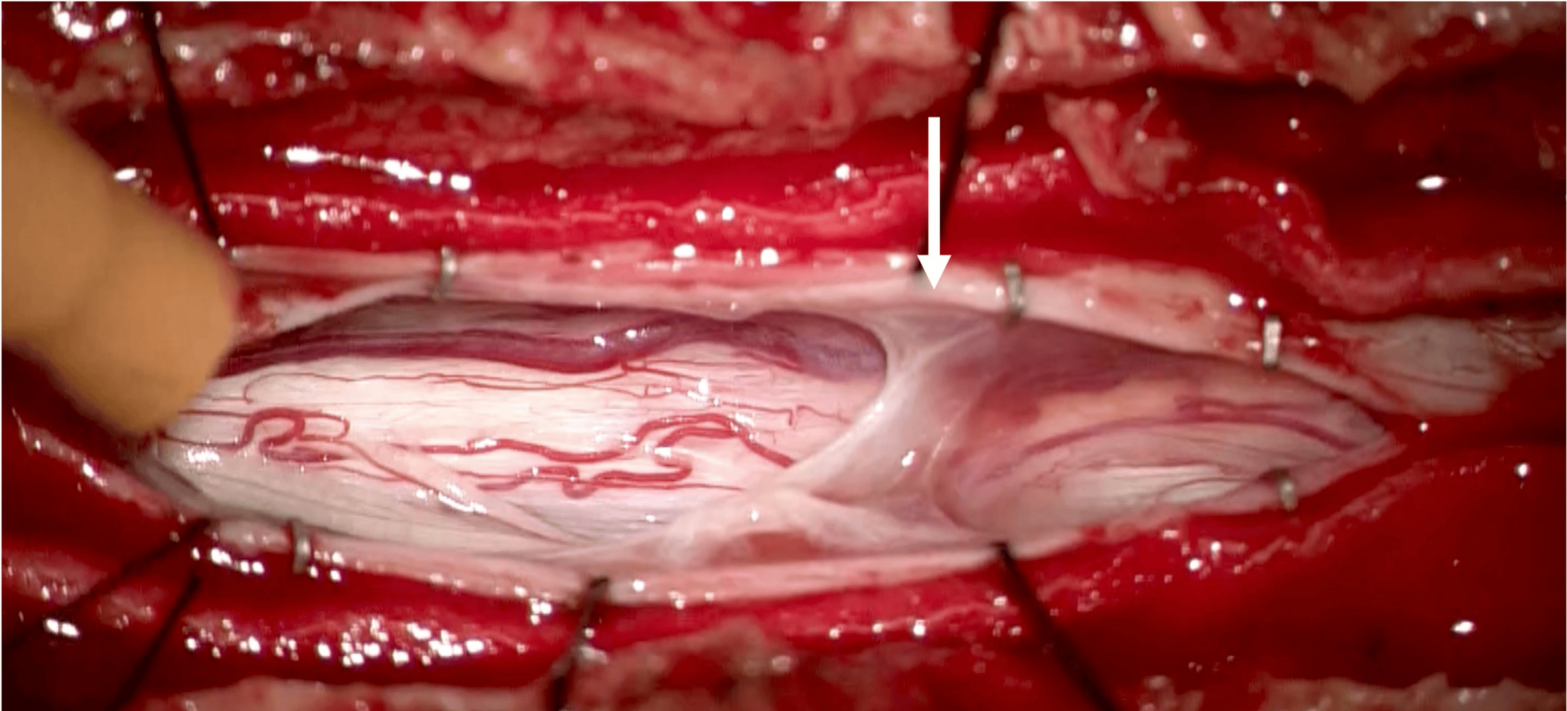
Intraoperative microscopic photo. On the left is the cephalad side. Incision through the dura and arachnoid revealed a scarred arachnoid tissue at the T6/7 level adhering to the posterior surface of the spinal cord and obstructing CSF perfusion (white arrow: arachnoid web).

Following the incision of the dura mater, the arachnoid membrane was scarred at the T7 level and adhered to the dorsal spinal cord, consequently obstructing the flow of CSF in the corresponding area (Figure 4). The arachnoid web adhering to the dorsal spinal cord was resected as far as possible laterally with micro scissors, and uncompromised arachnoid and dura mater were sutured in layers. Postoperatively, the patient demonstrated early improvement in lower extremity muscle strength, was able to ambulate with a cane, and exhibited resolution of right lower leg temperature and pain sensory disturbances. However, numbness in the lower extremities and bowel and bladder dysfunction persisted one year postoperatively.

## Discussion

SAW is an uncommon condition that often manifests as progressive myelopathy. Therefore, it is imperative to diagnose it promptly and offer suitable treatments. Here, two atypical findings of SAW were observed. First, the patient did not show the scalpel sign seen on MRI due to preoperative dorsal compression of the SAW, and he showed only spinal cord atrophy and intramedullary hyperintensities at the same site. Second, there were left-right differences in lower extremity neurological symptoms, with significant muscle weakness in the left and temperature and pain sensory disturbances in the right lower extremity.

Regarding the diagnosis of SAW, Reardon et al. have primarily described characteristic MRI findings, with a well-known sign being the so-called "scalpel sign" [7]. This sign, observed in all cases of SAW as reported by Voglis et al., involves spinal cord deformation due to dorsal compression by the SAW, which results in the appearance of a scalpel blade [3]. Additionally, a syrinx was present in 83% of patients. Consistent with these observations, systematic reviews have corroborated the scalpel sign as a hallmark feature of SAW. A comprehensive review by Vattipally et al. further substantiated the prevalence of the scalpel sign and noted its presence in 98% of cases [10]. Carr et al.'s systematic investigation of 127 SAW cases further refined the classification system by dividing the cases into three distinct groups based on MRI findings: type 1, spinal cord deformity only; type 2, cord deformity with myelomalacia; type 3, cord deformity with myelomalacia and syringomyelia. The findings showed that 54% of cases were classified as type 1, indicating that all cases of SAW resulted in some degree of spinal cord deformation [2].

In the present case, although spinal cord atrophy was observed, the characteristic scalpel sign was absent, rendering the MRI findings atypical and consequently challenging the definitive diagnosis of SAW before surgery. However, in the present case, a flow void was identified on MRI on the dorsal aspect of the spinal cord. Flow void represents a form of MRI artifact that refers to signal loss in moving fluids (usually blood, but also commonly observed in CSF or urine). Consequently, the presence of a flow void within a blood vessel is indicative of vascular patency. In the present case, the flow void was identified on the dorsal side of the flattened spinal cord on MRI. When considered in conjunction with the intraoperative ultrasound findings, it is hypothesized that the CSF colliding with the SAW at the same site caused a rapid change in CSF flow, which manifested as a flow void. Given the fact that SAW invariably causes CSF flow obstruction, a dorsal flow void may serve as a finding suggestive of SAW.

The patient presented with significant left-sided muscle weakness and temperature and pain sensory disturbance in the right lower extremity, which manifested as the Brown-Sequard syndrome. To the best of our knowledge, there have been no previous reports of similar symptoms. The most common symptoms of SAW are those associated with thoracic myelopathy, such as lower-extremity muscle weakness, gait disturbance, and numbness and pain in the lower extremities. Intraoperative ultrasonography is effective in the diagnosis of SAW and the evaluation of resection [9,10]. In this case, the septum posticum was seen to swing from side to side in the horizontal section, suggesting a left-right difference in the CSF perfusion in the posterior spinal cord. The left spinal fluid pressure was likely elevated relative to that of the right, resulting in a greater impact on the left side of the spinal cord. Postoperatively, there were signs of improvement in muscle strength, and the patient's right lower extremity temperature and pain sensory disturbances improved. Thus, SAWs can cause spinal cord pressure elevation due to impaired CSF flow from the posterior spinal cord, and the left-right difference in symptoms may be caused by the left-right difference in CSF pressure divided by the septum posticum during spinal cord compression. Brown-Sequard syndrome often occurs in cases of spinal cord herniation, and a differential diagnosis is necessary.

The etiology of SAW remains to be fully elucidated, but a previous multicenter study reported that 16% of patients with SAW had a history of previous spinal surgery. Conversely, most patients who underwent spinal surgery did not develop SAW, rendering the association uncertain [1]. In this case, the patient underwent lumbar endoscopic decompression surgery at another hospital, but details such as whether there was intraoperative dural injury were unknown, and it had been more than 10 years since the surgery, making an association unlikely.

The primary treatments for SAW are laminectomy and resection of the SAW [1,2,6,11]. A systematic review by Nisson et al. reported improvements in neurological symptoms in 91% of cases [1]. However, there are reports that surgery is unnecessary [10], and Carr et al. found that many cases of spinal deformity alone, i.e., without myelomalacia or a syrinx, do not require surgical intervention [2]. In the present case, the presence of myelomalacia in the spinal cord, as well as the patient's exhibited gait disturbance, necessitated surgical intervention. However, owing to the prolonged duration of the disease, some neurological symptoms persisted postoperatively. These findings emphasize the importance of an early and accurate diagnosis in the management of SAW.

## Conclusions

The diagnosis of SAW is challenging, and the characteristic MRI findings include the scalpel sign and spinal deformities. The scalpel sign is observed in most patients; however, even in cases where it is not evident, if there are findings indicative of spinal cord compression and a flow void behind the spinal cord, SAW should be suspected, and surgical intervention may be considered. Notably, SAW has the potential to manifest as Brown-Sequard syndrome, as evidenced in the present case. The underlying pathophysiology is presumed to be attributable to the septum posticum, which generates a disparity in CSF pressure between the left and right sides of the dorsal spinal cord.
